# Weighted Least Squares Techniques for Improved Received Signal Strength Based Localization

**DOI:** 10.3390/s110908569

**Published:** 2011-09-02

**Authors:** Paula Tarrío, Ana M. Bernardos, José R. Casar

**Affiliations:** Data Processing and Simulation Group, Universidad Politécnica de Madrid, ETSI. Telecomunicación, Avda. Complutense 30, 28040 Madrid, Spain; E-Mails: abernardos@grpss.ssr.upm.es (A.M.B.); jramon@grpss.ssr.upm.es (J.R.C.)

**Keywords:** localization, positioning, wireless networks, least squares, received signal strength, channel model estimation

## Abstract

The practical deployment of wireless positioning systems requires minimizing the calibration procedures while improving the location estimation accuracy. Received Signal Strength localization techniques using propagation channel models are the simplest alternative, but they are usually designed under the assumption that the radio propagation model is to be perfectly characterized a priori. In practice, this assumption does not hold and the localization results are affected by the inaccuracies of the theoretical, roughly calibrated or just imperfect channel models used to compute location. In this paper, we propose the use of weighted multilateration techniques to gain robustness with respect to these inaccuracies, reducing the dependency of having an optimal channel model. In particular, we propose two weighted least squares techniques based on the standard hyperbolic and circular positioning algorithms that specifically consider the accuracies of the different measurements to obtain a better estimation of the position. These techniques are compared to the standard hyperbolic and circular positioning techniques through both numerical simulations and an exhaustive set of real experiments on different types of wireless networks (a wireless sensor network, a WiFi network and a Bluetooth network). The algorithms not only produce better localization results with a very limited overhead in terms of computational cost but also achieve a greater robustness to inaccuracies in channel modeling.

## 1. Introduction

The capability of gathering enough data from the environment and the users enables the existence of intelligent spaces, which are able to process the collected information in order to provide useful services or information. Intelligent spaces often feature a collection of sensors and sensors networks, which collect the required information (e.g., environmental parameters such as temperature or humidity, biometric information from personal sensors, *etc*.), and actuators and robots, which perform the appropriate actions. Knowing the position of the sensors and the robots is fundamental to contextualize the information gathered by the sensors and to control the robots in an efficient way. Some of the sensors may be mobile, as they could be associated with mobile objects or people. Furthermore, the robots may not be equipped with self-navigation techniques. As a consequence, the availability of robust, accurate and easily deployable location systems is a key enabler of intelligent spaces and still an open challenge.

Although several technologies can be used to estimate the position of the different objects [1] (ultrasounds, artificial vision, infrared, GPS, *etc*.), radiofrequency localization techniques [2] have become very popular and suitable for this kind of sentient spaces, as they reuse the wireless infrastructure. Location may be computed from different parameters, such as time-of-flight, angle of arrival or received signal strength (RSS). Nevertheless, only the latter parameter is feasible in most commercial wireless technologies without hardware or software modifications. As the RSS information can be easily collected with off-the-shelf equipment, it has become the basis for the most popular techniques for inferring the relative positions of the nodes in the wireless network.

In the literature, two main approaches have been proposed to solve the localization problem using RSS measurements: channel modeling based methods and fingerprint strategies. In the first one [3–9], a propagation channel model is used to establish a relation between the RSS and the distance between two nodes. The location of a node can then be determined from a set of these distances using some positioning algorithm, such as the ones in [10] or [11]. Conversely, the second approach [9,10,12–15] creates a radio map of the environment by gathering, for each node, a set of RSS measurements in different positions, uniformly spaced on a regular grid. These “fingerprints” are then stored in a database; when an unknown node needs to be localized, its RSS measurements are matched against the ones stored in the map in order to find the closest correspondence. The main drawback of this approach is that a large number of on site measurements are required in order to obtain fine-grained localization; this situation unavoidably entails an increase of the operational cost. Additionally, fingerprint methods require an exhaustive, periodic and non-reusable preliminary calibration phase, which is usually infeasible in practical deployments.

With respect to channel model based techniques, they are built on the fact that a channel model is a theoretical, simplified and non-perfect approach to describe the behavior of a complex propagation environment. The model-based localization approach entails a much simpler calibration phase, as it only requires the calculation of the channel model, which can be estimated from a few measurements in the deployment area or even established theoretically without the need of a previous measurement phase. The more effort is put into the calibration, the better accuracy is obtained in the localization results, as the channel model will be better adapted to the particularities of the real propagation environment. But ideally, the calibration processes should be minimized in order to make the system deployment easier and less time consuming. Therefore, a solution to these inconvenient calibration needs is the design of positioning algorithms that are robust to the inaccuracies in the channel estimation; otherwise said, strategies capable of obtaining accurate location estimates in spite of working on non-accurately calibrated channel models.

In this paper we propose and evaluate the use of two weighted least squares techniques to calculate the position of a mobile node from the estimated distances to some reference nodes. The standard RSS-based localization techniques for wireless networks do not consider the individual accuracies of the different measurements to construct a better estimator. The proposed algorithms aim at enhancing the accuracy of position estimates while reducing their sensitivity to an imperfectly modeled channel. Although weighted least squares techniques are very well-known, to our knowledge the application of these techniques to the RSS-based localization problem and, in particular, to make localization more robust to imperfect channel models, has not been presented in detail before. Our work includes an exhaustive analysis based on both simulated and empirical tests, which shows that the location results are not only more accurate, as expected for a weighting technique, but also more robust to channel estimation errors. As explained above, this fact makes these techniques very attractive from a practical point of view.

The structure of the paper is as follows. In Section 2 the related state of the art is reviewed and in Section 3 the fundamentals of channel model based localization methods are described. Sections 4 and 5 describe the proposed positioning algorithms, the weighted hyperbolic technique and the weighted circular positioning technique. Section 6 includes a performance analysis of the proposed methods with numerical simulations and Section 7 analyzes the performance of the methods through real experiments with three different wireless networks: a WiFi network, a wireless sensor network and a Bluetooth network. With this experimental validation we show that the proposed techniques reduce the localization error with respect to the standard hyperbolic and circular positioning algorithms and that they have a bigger robustness to inaccuracies in the channel estimation. We also analyze the computational load of the algorithms, an issue which may be critical when considering embedded implementations. Section 8 concludes the paper.

## 2. Related Work

The problem of range-based localization has been studied for many years, especially in the field of radar and sonar, where the range measurements are usually obtained from time of arrival (TOA) or time difference of arrival (TDOA) measurements, and more recently in cellular networks, motivated by the FCC E-911 norm. In these areas, weighting techniques have been previously used to solve TOA/TDOA-based localization problems. For example, in [16] a weighted least squares estimator that achieves a better accuracy than the standard least squares estimator is used to calculate the position of a mobile phone from TOA measurements.

In ad hoc and sensor networks, the position of the nodes is typically computed from RSS measurements, which are then converted into distances using a channel model. This problem is in fact a range-based localization problem; however, very few works have studied the use of weighting techniques for RSS-based localization. The authors of [17] propose a distributed weighted multidimensional scaling algorithm to determine the position of a node in a sensor network by minimizing a global cost function in which each distance measurement is weighted by a different factor. The authors suggest that the weights should be selected to reflect the accuracy of the measurements, but adopt a weighting scheme independent from the channel model. In [18] different weighting schemes for the multidimensional scaling formulation are proposed and compared.

However, these and most of the existing works in RSS-based channel modeling localization consider that the radio propagation model is known a priori, either because a certain model is assumed (for example, perfect free-space propagation) or because the parameters of the channel model are supposed to be estimated in the deployment environment prior to the real operation of the system. The first assumption is not realistic, as the propagation conditions in the real scenario may differ from those predicted by the theoretical model. On the other hand, taking measurements in the deployment area to estimate the channel model is not always possible (for example, in hostile environments) and, if possible, the number of measurements that can be taken is limited, thus the estimated model is usually a poor representation of the real channel. Consequently, to be applied in a real deployment a localization technique should take into consideration these concerns, either by trying to model the environment in a more realistic way or by using a positioning technique that is more robust to the model inaccuracies.

Few studies have been carried out to develop techniques in which the channel model is calculated during localization, or periodically updated, in order to reduce the localization errors that are produced by the inaccuracies of the propagation model. A common approach leverages RSS measurements between reference nodes with known positions to periodically estimate updated channel models and avoid using old models that no longer represent the channel behavior. These updated channels may also be different depending on the position of the nodes. In this way, these techniques use channel models that try to be consistent with the actual propagation characteristics. Some examples of localization algorithms using this approach can be found in [19–21]. Although these varying models typically characterize the real propagation behavior with greater accuracy than fixed models, they are usually updated from a limited set of noisy measurements, therefore, the estimation of the channel parameters is still not perfect.

Another approach consists in assuming that the radio propagation is characterized by a given model and estimate the value of the channel parameters together with the position of the mobile nodes. For example, in [6] one of the parameters of the channel model is estimated together with the position of the node using a non-linear least squares estimator, and in [22] the two parameters of the channel model are estimated together with the target position by means of an iterative maximum likelihood estimator. In [23] a tunable parametric channel model is used to obtain the position of a mobile node with greater accuracy than using a fixed channel model. Nevertheless, a deep understanding of the effects of channel estimation errors on the localization results is still missing. In this paper, we specifically consider and describe the problem of using inaccurate channel models for localization and propose two weighted least squares techniques that achieve a greater robustness to channel model inaccuracies than standard RSS-based positioning techniques.

## 3. Channel Modeling Localization

In this section we briefly describe a typical localization method based on channel modeling. Let us consider a wireless network composed of both mobile and fixed (or anchor) nodes. In an ad hoc network, these fixed nodes are ordinary nodes that are chosen for this purpose. In a WiFi network, these nodes could be the access points. The position of a mobile node can be calculated using the model-based approach in the following way. First, the mobile node measures the RSS received from the anchor nodes. Second, a channel model is used to estimate, from the RSS measurements, the distances between the mobile node and each anchor node. And finally, the position of the node is determined from these distances using a multilateration algorithm.

Many channel models have been proposed for outdoor and indoor environments [24]. The most popular channel model for RSS-based localization, due to its simplicity, is the lognormal shadowing path loss model [25], but other models (Nakagami fading model, Rayleigh fading, Ricean fading, *etc*.) have been also used [3,4,7]. Any channel model allows estimating the distance between nodes from the received signal strength. For example, the relation between the received power (*P_RX_*) and the distance (*d*) between transmitter and receiver for the lognormal channel model is given by

(1) $$P_{\mathrm{RX}}(\mathrm{dBm})=A-10\eta \;\text{log}\frac{d}{d_{0}}+N$$

where *A* is a constant term, *η* is the path loss exponent, and *N* ∼ 𝒩 (0, *σ*^2^) is a zero-mean gaussian random variable with standard deviation *σ*. The constant term *A* depends on the transmission power *P_TX_*, on the transmitter’s and receiver’s antenna gains and on the power loss for a reference distance *d*_0_, and has to be experimentally determined. On the other hand, the path loss exponent *η* typically ranges between 2 and 4 depending on the environment, and it has to be experimentally determined too.

In a real localization application, the radio channel has to be either predicted theoretically or, in the best case, estimated from a limited and usually small set of RSS measurements. As a result, the estimated model, characterized for example by the parameters *η* and *A*, may not be an accurate representation of the real radio channel of the environment. This will introduce errors in the RSS to distance conversion, producing in the end worse localization results.

As said before, once the distances to different anchor nodes are estimated, a positioning algorithm must be applied in order to calculate the position of the mobile node. Two of the simplest and most common positioning algorithms that have been used for RSS-based localization are the circular positioning algorithm [6,11] and the hyperbolic positioning algorithm [11,26]. The basic idea of the circular positioning algorithm is to find the position (*x*, *y*) of the mobile node that minimizes the sum of the squared errors in the set of estimated distances. If (*x_i_*, *y_i_*) is the position of anchor node *i* (*i* = 1, 2, . . . , *N*, where *N* is the number of anchor nodes) and *d̃_i_* is the distance estimation to anchor node *i*, this error is given by

(2) $$ɛ=\sum_{i=1}^{N}{\left(\sqrt{{\left(x_{i}-x\right)}^{2}+{\left(y_{i}-y\right)}^{2}}-{\tilde{d}}_{i}\right)}^{2}$$

The position (*x*, *y*) of the mobile node can be then calculated iteratively using for example a straight gradient method

(3) $${\left[\begin{array}{c} \hat{x}\\ \hat{y} \end{array}\right]}_{k+1}={\left[\begin{array}{c} \hat{x}\\ \hat{y} \end{array}\right]}_{k}-\alpha {\left[\begin{array}{c} \frac{\partial ɛ}{\partial x}\\ \frac{\partial ɛ}{\partial y} \end{array}\right]}_{x={\hat{x}}_{k},y={\hat{y}}_{k}}$$

This method requires an initial location estimation.

The hyperbolic positioning algorithm converts this problem into a linear problem that can be solved with a least squares estimator, as we explain next. Using the previous notation, the square of the distance between the mobile node and anchor node *i* can be expressed as

(4) $$d_{i}^{2}={\left(x_{i}-x\right)}^{2}+{\left(y_{i}-y\right)}^{2}$$

Without loss of generality, the origin of coordinates can be taken at anchor node *i* = 1, that is, *x*_1_ = *y*_1_ = 0. Thus, for *i* > 1

(5) $$d_{i}^{2}-d_{1}^{2}=x_{i}^{2}+-2xx_{i}+y_{i}^{2}-2yy_{i}$$

Expressing Equation (5) in matrix form

(6) $$\left[\begin{array}{cc} 2x_{2} & 2y_{2}\\ \vdots & \vdots \\ 2x_{N} & 2y_{N} \end{array}\right]\left[\begin{array}{c} x\\ y \end{array}\right]=\left[\begin{array}{c} x_{2}^{2}+y_{2}^{2}-d_{2}^{2}+d_{1}^{2}\\ \vdots \\ x_{N}^{2}+y_{N}^{2}-d_{N}^{2}+d_{1}^{2} \end{array}\right]$$

In the case of RSS-localization, we do not know the real distances *d_i_* between mobile and anchor nodes. Instead, we have some noisy estimations *d̃_i_*. Therefore, our problem can be formulated as

(7) $$H\cdot \bar{x}=\tilde{b}$$

where $H=\left[\begin{array}{cc} 2x_{2} & 2y_{2}\\ \vdots & \vdots \\ 2x_{N} & 2y_{N} \end{array}\right]$, $\bar{x}=\left[\begin{array}{c} x\\ y \end{array}\right]$ and *b̃* is a random vector given by

(8) $$\tilde{b}=\left[\begin{array}{c} x_{2}^{2}+y_{2}^{2}-{\tilde{d}}_{2}^{2}+{\tilde{d}}_{1}^{2}\\ \vdots \\ x_{N}^{2}+y_{N}^{2}-{\tilde{d}}_{N}^{2}+{\tilde{d}}_{1}^{2} \end{array}\right]$$

Therefore, the position of the mobile node can be calculated as the least-squares solution of this equation, given by

(9) $$\hat{x}={\left(H^{T}H\right)}^{-1}H^{T}\tilde{b}$$

Note that this hyperbolic algorithm does not directly minimize the error given by Equation (2), but a non-linear function of it. Therefore, the performance of this algorithm is expected to be worse than that of the circular algorithm.

These two typical positioning algorithms used for RSS-based localization give the same weight to the different distance estimations. But, since the RSS does not depend linearly on the distance between the nodes, the same error in the RSS measurement will produce larger errors in the distance estimation if the distance between the nodes is higher, as it can be deduced from Equation (1). That is, the accuracy of the distance estimations depends on the distance itself. Therefore, by giving more weight to those measurements which have a greater accuracy, that is, the measurements corresponding to short distances, we obviously expect to obtain a greater accuracy in the localization result. This observation led us to propose the use of two weighted techniques to improve the accuracy of the hyperbolic and circular positioning algorithms respectively. But what is more interesting is that these weighted techniques are also more robust to errors in the estimation of the channel parameters, as we will show in Sections 6 and 7. This is an especially desirable characteristic when deploying an operative location system, as it is never convenient to accomplish complicated and time consuming calibration processes. In these situations, using a theoretical or roughly calibrated channel model can provide sound estimates if the location algorithms are sufficiently robust to deal with imperfect models, as it occurs with the proposed algorithms following described.

## 4. Weighted Hyperbolic Algorithm

The linear problem in Equation (7) can be solved using a weighted least-squares estimator, as proposed in [27]

(10) $$\hat{x}={\left(H^{T}S^{-1}H\right)}^{-1}H^{T}S^{-1}\tilde{b}$$

where *S* is the covariance matrix of vector *b̃*. Note that the noise affecting the measurement vector *b̃* does not have zero mean, so the estimator Equation (10) is biased. Assuming that the measurements of the distances *d̃_i_* to different reference nodes are independent and as *x_i_* and *y_i_* are constants, the matrix *S* can be easily calculated. From Equation (8)

(11) $$S=\left[\begin{array}{cccc} \mathrm{Var}({\tilde{d}}_{1}^{2})+\mathrm{Var}({\tilde{d}}_{2}^{2}) & \mathrm{Var}({\tilde{d}}_{1}^{2}) & \ldots & \mathrm{Var}({\tilde{d}}_{1}^{2})\\ \mathrm{Var}({\tilde{d}}_{1}^{2}) & \mathrm{Var}({\tilde{d}}_{1}^{2})+\mathrm{Var}({\tilde{d}}_{3}^{2}) & \ldots & \mathrm{Var}({\tilde{d}}_{1}^{2})\\ \vdots & \vdots & \ddots & \vdots \\ \mathrm{Var}({\tilde{d}}_{1}^{2}) & \mathrm{Var}({\tilde{d}}_{1}^{2}) & \ldots & \mathrm{Var}({\tilde{d}}_{1}^{2})+\mathrm{Var}({\tilde{d}}_{N}^{2}) \end{array}\right]$$

where *Var* stands for variance. The terms of the covariance matrix *S* can be calculated as

(12) $$\mathrm{Var}\left({\tilde{d}}_{i}^{2}\right)=E\left[{\tilde{d}}_{i}^{4}\right]-{\left(E\left[{\tilde{d}}_{i}^{2}\right]\right)}^{2}$$

Assuming that the channel is lognormal, it can be rapidly derived from Equation (1) that the estimated distance *d̃_i_* is a random variable defined by

(13) $${\tilde{d}}_{i}=d_{i}\cdot 10^{\frac{N(0,\sigma )}{10\eta }}=10^{N({\text{log}}_{10}\;d_{i},\frac{\sigma }{10\eta })}=e^{N({\text{log}}_{10}\;d_{i},\frac{\sigma }{10\eta })\;\text{ln 10}}=e^{N(\text{ln}d_{i},\frac{\sigma \;\text{ln}\;10}{10\eta })}$$

that is, *d̃_i_* is a lognormal random variable with parameters *μ_d_* = ln *d_i_* and $\sigma_{d}=\sigma \cdot \frac{\text{ln}\;10}{10\eta }$. The k-th moment of a lognormal random variable of parameters (*μ_d_*, *σ_d_*) is given by $\mu_{k}=e^{k\cdot \mu_{d}+\frac{k^{2}\sigma_{d}^{2}}{2}}$. Therefore

(14) $$E\left[{\tilde{d}}_{i}^{4}\right]=\text{exp}\;(4\mu_{d}+8\sigma_{d}^{2})$$

(15) $$E\left[{\tilde{d}}_{i}^{2}\right]=\text{exp}\;(2\mu_{d}+2\sigma_{d}^{2})$$

Finally, substituting these values into Equation (12), we obtain the following expression for the terms of the covariance matrix *S*

(16) $$\mathrm{Var}\left({\tilde{d}}_{i}^{2}\right)=E\left[{\tilde{d}}_{i}^{4}\right]-{\left(E\left[{\tilde{d}}_{i}^{2}\right]\right)}^{2}=\text{exp}\;(4\mu_{d})\cdot (\text{exp}\;(8\sigma_{d}^{2})-\text{exp}(4\sigma_{d}^{2}))$$

It should be noted that *μ_d_* depends on the real distance *d_i_* between the mobile and the anchor nodes. Therefore, in order to use the estimator in Equation (10) in a real deployment, it is necessary to approximate the real distance *d_i_* by the estimated distance *d̃_i_*. As the value of *σ_d_* is constant, that is, it is the same for every distance estimation, the factor $\text{exp}(8\sigma_{d}^{2})-\text{exp}(4\sigma_{d}^{2})$ can be taken out from matrix *S* as a common factor and, therefore, its value does not affect the estimated position according to Equation (10). Consequently, the value of the parameter *σ* of the channel model does not need to be estimated in order to apply this positioning technique. Taking these two observations into account, the terms of the covariance matrix can be calculated using

(17) $$\mathrm{Var}\left({\tilde{d}}_{i}^{2}\right)={\tilde{d}}_{i}^{4}$$

This weighted technique involves the computation of the inverse of matrix *S*, therefore its computational cost is O(*n*^3^), where *n* is the number of reference nodes inside the coverage area of the mobile node (and not the total number of reference nodes). On the other hand, the classical hyperbolic position algorithm has an asymptotic cost of O(*n*), since it only involves matrix multiplications. Therefore, although the weighted algorithm is expected to produce better localization results, it is also more expensive from a computational point of view, especially when *n* is high. However, in practical deployments the value of *n* is usually small, so both hyperbolic methods can be executed in practice in resource-constrained devices.

## 5. Weighted Circular Algorithm

The second technique that we consider is the weighted circular algorithm, which is based on the circular positioning technique but introduces a different weight for each measurement. The basics of the algorithm are explained next. The error in the distance estimation for anchor node *i* is given by

(18) $$e_{i}=d_{i}-{\tilde{d}}_{i}=\sqrt{{\left(x_{i}-x\right)}^{2}+{\left(y_{i}-y\right)}^{2}}-{\tilde{d}}_{i}$$

In order to estimate the position (*x*, *y*) of the mobile node, we consider the minimization of the weighted least squares error criterion, which is given by the following expression

(19) $$ɛ=e^{T}S^{-1}e$$

where *S* is the covariance matrix of the random vector *e* = (*e*_1_ *e*_2_ … *e_N_*)*^T^*. Assuming that the errors *e_i_* in the measurements of the distances to different reference nodes are independent, *S* is a diagonal matrix with diagonal elements [*S*]*_ii_* = *Var*(*e_i_*). Thus, the error to be minimized is

(20) $$ɛ=eS^{-1}e^{T}=\sum_{i=1}^{N}\frac{e_{i}^{2}}{\mathrm{Var}(e_{i})}=\sum_{i=1}^{N}\frac{1}{\mathrm{Var}({\tilde{d}}_{i})}{\left(\sqrt{{\left(x_{i}-x\right)}^{2}+{\left(y_{i}-y\right)}^{2}}-{\tilde{d}}_{i}\right)}^{2}$$

where the variance of *d̃_i_* can be calculated as

(21) $$\mathrm{Var}\left({\tilde{d}}_{i}\right)=E\left[{\tilde{d}}_{i}^{2}\right]-{\left(E\left[{\tilde{d}}_{i}\right]\right)}^{2}=\text{exp}\;(2\mu_{d})\cdot (\text{exp}\;(2\sigma_{d}^{2})-\text{exp}\;(\sigma_{d}^{2}))$$

As it can be noticed, the function that we want to minimize in this case (Equation (20)) is very similar to the function that is minimized in the circular algorithm (Equation (2)), except from some weighting factors that emphasize the contribution of those distance measurements that are expected to be more reliable, *i.e*., those with a smaller variance.

The position (*x*, *y*) of the mobile node can be then calculated iteratively using, for example, a straight gradient method (Equation (3)) for minimizing Equation (20).

As the value of *σ_d_* is constant, that is, it is the same for every distance estimation, the factor $\text{exp}\;(2\sigma_{d}^{2})-\text{exp}\;(\sigma_{d}^{2})$ can be taken out in Equation (20) as a common factor that multiplies all the terms of the sum and, therefore, its value does not affect the estimated position. As a result, the value of the parameter *σ* of the channel model does not need to be estimated in order to apply this positioning technique.

This technique is expected to perform better than the standard ones and than the weighted hyperbolic technique, as it considers the accuracy of the distance measurements and it is based on the circular algorithm (which gives better localization results than the hyperbolic algorithm, as it minimizes directly the distance errors and not some non-linear function of them as in the hyperbolic algorithm). But on the other hand, it is an iterative method, so its computational load may be greater.

The computational complexity of both circular positioning algorithms (classical and weighted) is O(*I* · *n*), where *I* is the number of iterations needed for convergence and *n* is the number of reference nodes inside the coverage area of the mobile node. Although they have the same asymptotic complexity, the weighted circular algorithm is a little more complex, since it involves the computation of the weighting factors. On the other hand, compared with the classical hyperbolic algorithm whose asymptotic complexity is O(*n*), the circular algorithms are more complex due to their iterative nature. Finally, given that the complexity of the weighted hyperbolic algorithm is O(*n*^3^), we expect a smaller computational load for this algorithm when the value of *n* is small, whereas for high values of *n* the circular algorithms would be a better choice.

In the following sections we show the performance of the two weighted techniques in comparison to the standard hyperbolic and circular positioning techniques for RSS-based localization. This comparison is made both with simulated RSS data and experimental data from a real sensor network deployment, from a WiFi deployment and from a Bluetooth deployment.

## 6. Performance Results for Simulated Data

In this section we evaluate through some simulations in Matlab the performance of the proposed positioning techniques in terms of the accuracy of the localization results. We compare these results with those obtained with the standard hyperbolic and circular positioning algorithms and with the Cramer-Rao lower bound. We pay a particular attention to the robustness of these techniques to errors in the estimated channel model, that is, on how the accuracy degrades as the estimated channel model differs from the optimum one. The more robust the technique is, the more attractive from the practical point of view it will be, since it will behave better in a real situation where the channel is estimated from a limited set of measurements.

We also study the computational load of the considered algorithms, which is an important factor for two reasons. On the one hand, using algorithms with low computational cost is usually interesting, especially in resource-constrained devices in which the processing capacity is quite limited. On the other hand, the computational load is an indicator of the energy that will be consumed in the computation of the algorithm. This is an important issue if the algorithm is executed in the nodes and not in a central processor, since in wireless devices energy is a limited resource that must be saved and, although the transmission and reception of radio signals is the most energy consuming task in wireless devices, the computation also contributes to the energy consumption.

The Cramer–Rao lower bound for the localization problem with RSS measurements is derived in [28]. Assuming the lognormal model given by Equation (1), the Fisher information matrix for a network composed of *N* reference nodes and one mobile node with unknown position is given by

(22) $$F=\left[\begin{array}{cc} F_{\mathrm{xx}} & F_{\mathrm{xy}}\\ F_{\mathrm{xy}} & F_{\mathrm{yy}} \end{array}\right]$$

where

(23) $$F_{\mathrm{xx}}=b\sum_{i=1}^{N}\frac{{\left(x-x_{i}\right)}^{2}}{d_{i}^{4}},\;F_{\mathrm{yy}}=b\sum_{i=1}^{N}\frac{{\left(y-y_{i}\right)}^{2}}{d_{i}^{4}},\;F_{\mathrm{xy}}=b\sum_{i=1}^{N}\frac{\left(x-x_{i})(y-y_{i}\right)}{d_{i}^{4}}$$

$$b={\left(\frac{10\eta }{\sigma \;\text{ln }10}\right)}^{2}$$

and the Cramer–Rao bound is given by

(24) $$\sigma^{2}=E[{\left(\tilde{x}-x\right)}^{2}+{\left(\tilde{y}-y\right)}^{2}]\geq \frac{F_{\mathrm{xx}}+F_{\mathrm{yy}}}{F_{\mathrm{xx}}F_{\mathrm{yy}}-F_{\mathrm{xy}}^{2}}$$

Note that the Cramer–Rao bound indicates the minimum variance an unbiased estimator of the position can achieve, thus, it is not directly comparable with the Mean Square Error (MSE) of the proposed estimators, which are biased. However, in the following figures representing the MSE of the proposed algorithms, we also show as a reference the Cramer–Rao bound.

The simulation environment is the following. We consider a 100 m × 100 m room, with *N* anchor nodes and one mobile node that is situated randomly throughout the room. In each position of the mobile node, the *N* RSS values (one for each anchor node) are simulated using the lognormal channel model (Equation (1)) with *η* = 3, different values of *A* (which simulates different coverage areas) and different values of *σ*. When the simulated RSS is below the receiver sensitivity (we have set this sensitivity to −96 dBm, as in the MicaZ motes [29]), we simulate that the measurement is not available. These RSS values are then converted to estimated distances through the channel model, given by Equation (1). It should be noticed that the *channel model* used for the RSS-distance conversion (characterized by the parameters *η_estim_* and *A_estim_*) does not have to be the same as the *real channel* (characterized by the parameters *η_real_* and *A_real_*). Indeed, in a practical deployment, the channel model is only an approximation to the real characteristics of the channel. We assume first that the parameters of the channel model *η* and *A* are perfectly known (*η_estim_* = *η_real_* and *A_estim_* = *A_real_*) and afterward we will show how the performance is affected when these parameters are estimated with some errors, *i.e.*, when *η_estim_* ≠ *η_real_* or *A_estim_* ≠ *A_real_*. Finally, using the known positions of the *N* anchor nodes and the distance estimates to them, the proposed positioning algorithms are applied in order to estimate the position of the mobile node.

### 6.1. Performance for Perfect Channel Models

**Figure 1.**
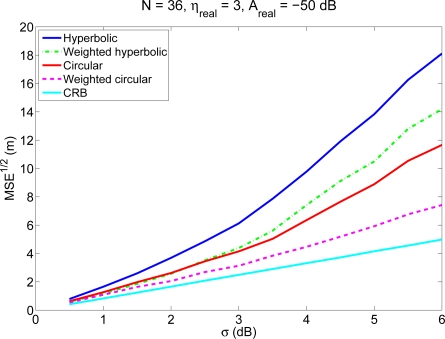
Average localization error (calculated as the square root of the MSE) as a function of the standard deviation *σ* of the RSS measurements for the four methods, for an environment with *N* = 36 anchor nodes (following a 20 m × 20 m grid) and *A_real_* = −50 dB (coverage radio around 30 m). The Cramer–Rao bound is shown as comparison.

We have carried out 1,000 simulations, varying randomly the positions of the mobile node throughout the room. Figure 1 shows the average localization errors as a function of the standard deviation *σ* of the RSS measurements for the four methods, for an environment with *N* = 36 anchor nodes (following a 20 m × 20 m grid) and *A_real_* = −50 dB (coverage radio around 30 m). The parameters of the channel model in this case were assumed to be perfectly known. It can be seen that the accuracy of the proposed methods is better than the accuracy obtained with the traditional algorithms, especially when the measurements of the RSS have a large standard deviation. A similar performance is obtained for other values of *N* and *A_real_*.

**Figure 2.**
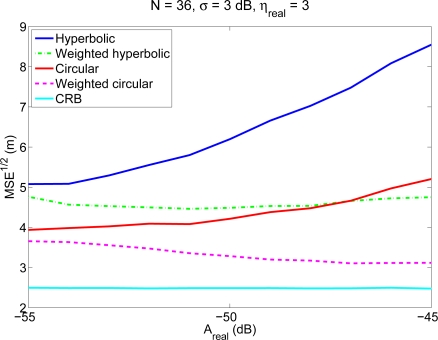
Average localization error (calculated as the square root of the MSE) of the four positioning methods as a function of *A_real_*, for an environment with *N* = 36 and *σ* = 3 dB. The Cramer–Rao bound is shown as comparison. The values of *n* corresponding to these values of *A_real_* vary between 3.9 and 14.2.

Figure 2 shows the average localization errors as a function of the parameter *A_real_* (which models in this case different coverage areas), for an environment with *N* = 36 anchor nodes (following a 20 m × 20 m grid) and *σ* = 3 dB. It should be noted that the variation with *A_real_* for a fixed value of *N* is equivalent to a variation of the number of reference nodes inside the coverage area of the mobile node (*n*). It can be seen that the performance of the traditional algorithms degrades when *A_real_* increases, that is, when the coverage area increases, as for a given node density this implies that the mobile node is able to receive signals from more distant reference nodes, that produce less reliable RSS measurements. On the contrary, weighted algorithms behave even better for bigger values of *A_real_*, that is, for higher coverage areas. This is due to the fact that the additional information coming from distant nodes, although less reliable, is weighted according to its uncertainty and becomes a valuable source of information which contributes to a better estimation of the position.

**Figure 3.**
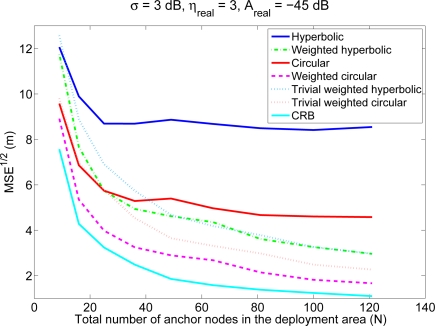
Average localization error (calculated as the square root of the MSE) of the four positioning methods as a function of *N*, for an environment with *A_real_* = − 45 dB and *σ* = 3 dB. The Cramer–Rao bound is shown as comparison. The values of *n* corresponding to these values of *N* vary between 6.2 and 50.

Figure 3 shows the average localization errors of the four positioning methods as a function of the number of anchor nodes, for an environment with *A_real_* = −45 dB (coverage radio around 45 m) and *σ* = 3 dB. Again, the dependency with *N* in this figure is equivalent to a dependency with the number of reference nodes inside the coverage area of the mobile node (*n*). The corresponding values of *n* in this figure range from 6.2 to 50, therefore, although we have included this large range to clearly see the tendencies, only the first part of the figure is interesting from a practical point of view. It can be seen that, for a fixed value of *A_real_*, the localization error diminishes when the number of anchor nodes increases, since the number of reliable RSS measurements (from close anchor nodes) increases with the anchor nodes density. The figure also shows, as comparison, two trivial weighting schemes that, based on the traditional hyperbolic and circular algorithms, just ignore lower power signals and therefore will have a lower computational cost than the weighted techniques proposed in this paper. These trivial weighting techniques get closer to the proposed weighted techniques as the number of reference nodes increases (or equivalently, as *n* increases). However, in real deployments the value of *n* is usually small, so in general, the proposed weighted techniques will have a better performance in terms of accuracy.

**Figure 4.**
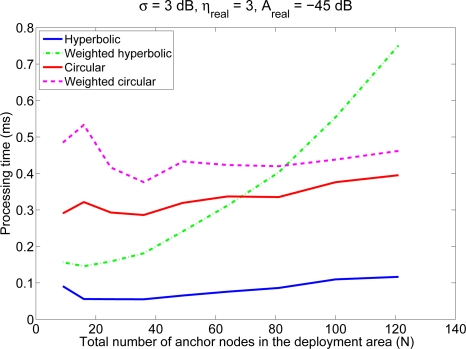
Average processing time of the different positioning algorithms as a function of the number of anchor nodes (*N*), for an environment with *A_real_* = −45 dB and *σ* = 3 dB. The corresponding values of *n* vary between 6.3 and 49.7.

Figure 4 shows the average processing time for the computation of the mobile node position as a function of the number of anchor nodes, for an environment with *A_real_* = −45 dB and *σ* = 3 dB. It should be noticed that although this parameter is dependent on the actual implementation of the algorithm and on the activities of the computer during the processing time, it can serve as a coarse indicator of the computational cost. Also, the processing time of the circular algorithms depend on the value of *α*, so in order to obtain a fair comparison, we have used in the simulations the best value of *α* for each of the algorithms (0.8 for the proposed weighted algorithm, 0.28 for the trivial weighting scheme and 1.9/*N* for the classical algorithm). Although the particular values in this figure are not significant, the relationship between the four algorithms and their dependency on the number of anchor nodes is relevant. The results indicate that the complexity of the circular positioning techniques and the classical hyperbolic technique grows linearly with *n*, whereas the complexity of the weighted hyperbolic algorithm grows faster, as expected. On the other hand, we can see that there is a crossover in complexity between the weighted hyperbolic algorithm and the circular algorithms: for small values of *n*, the hyperbolic technique is faster; for large values of *n* the circular algorithms are better. Anyway, in real deployments the value of *n* is usually small, so in general, the weighted hyperbolic algorithm will have a better performance in terms of complexity.

To sum up, the obtained results indicate that the accuracy of the proposed methods is better than the accuracy obtained with the traditional algorithms, especially when the measurements of the RSS have a large standard deviation and when the beacon density is high. We have also observed that the weighted circular technique has a very good performance and provides better localization results than the weighted hyperbolic technique, but at the expense of much higher computational costs (around three times the computational cost of the weighted hyperbolic techniques for typical (small) values of *n* in real deployments). From these results we can state that, for a certain application, if we need a great accuracy in the localization and we do not have energy constraints, we should probably use the weighted circular algorithm. On the other hand, if we have energy limitations we would have to consider the accuracy-energy consumption trade off to choose between the hyperbolic and the weighted hyperbolic techniques.

### 6.2. Performance for Imperfect Channel Models

In the previous simulations we have considered that the channel model used for the RSS-distance conversion was the same that the real channel model, that is, the channel model that was used to generate the RSS measurements. But in a real situation, the channel is usually characterized from a set of measurements and the estimated channel model may not be the best, that is, another model with slightly different parameters could lead to more accurate results. Therefore, it is very interesting to evaluate the sensitivity of the proposed algorithms to errors in the estimation of the channel parameters.

**Figure 5.**
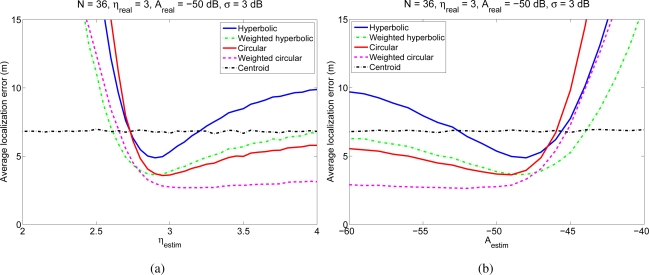
Average localization error for different positioning algorithms and for different values of the estimated parameters *η_estim_* and *A_estim_* of the channel model.

Considering the same simulation environment as in the previous experiment, that is, a 100 m × 100 m room, with *N* anchor nodes and one mobile node, we performed several simulations for different values of the parameters *η* and *A* of the *estimated channel model* (*η_estim_* and *A_estim_*), while the parameters of the *real channel* used to generate the RSS measurements in these simulations were fixed to *η_real_* = 3, *A_real_* = −50 dB and *σ* = 3 dB. For each value of the estimated *η_estim_* and *A_estim_* we carried out 1,000 simulations with different positions of the mobile node and *N* = 36. Figure 5(a) shows the average localization error as a function of the parameter *η_estim_* of the estimated channel, assuming that *A* is known without error (*A_estim_* = *A_real_*). Figure 5(b) shows the average localization error as a function of the parameter *A_estim_* of the estimated channel model assuming that *η* is known without error (*η_estim_* = *η_real_*). As a reference to highlight how quickly the performance degrades with an error in the channel estimation, we have included in the figures the localization errors of a straightforward positioning technique that estimates the position of the mobile node as the centroid of the positions of the reference nodes from which the mobile node receives signals. It can be seen that using the proposed or the classical positioning techniques with a wrongly estimated channel model can yield very poor localization results, even worse than those obtained with the simple centroid technique. Therefore, it is very important to estimate the parameters of the channel model with enough accuracy in order to achieve good position estimates. It is also interesting to note that if *η* is underestimated or *A* is overestimated, the performance of all the positioning techniques degrades very quickly, whereas for overestimated *η* and underestimated *A* the degradation is not so fast. This behavior is due to the fact that the channel model is logarithmic, so when *η* is underestimated or *A* is overestimated, the distances between the nodes are overestimated in a greater amount than when they are underestimated (when *η* is overestimated or *A* is underestimated). For example, if *A* is underestimated by an amount *X*, the estimated distance will be divided by a factor *α* = 10^*X*/10*η*^ > 1, whereas if *A* is overestimated by the same amount *X*, the estimated distance will be multiplied by the factor *α*. Clearly, the difference between the real and the estimated distances will be higher in the second case, so the localization error will be also higher in this case. A similar effect occurs if *η* is underestimated or overestimated. This fact should be taken into account when the parameters *A* and *η* are being estimated or chosen to model the channel. Finally, a remarkable aspect is that, although in all the cases the performance degrades when the estimated parameter differs from the real one, the two proposed weighted algorithms are less sensitive to these variations, and specially, the weighted circular technique, which is quite insensitive to overestimating *η* and underestimating *A*. This feature is very valuable in practical deployments, where the channel is usually roughly estimated.

## 7. Performance Results for Experimental Data

In this section we describe the results of some experimental tests to evaluate the accuracy, processing time and robustness of the proposed positioning algorithms in real wireless network deployments. Three wireless networks have been used for these experiments: a Wifi network, a Bluetooth network and a wireless sensor network composed of MicaZ motes. These deployments were used to collect real RSS measurements, which were later used to process the proposed algorithms off-line.

### 7.1. Description of the Experimental Deployments

The three experiments were carried out in an office area in which we deployed several reference nodes at different positions and a mobile node was used to test the algorithms. There were walls and furniture in the testing area that introduced non-line-of-sight propagation paths between the reference nodes and the mobile node.

**Figure 6.**
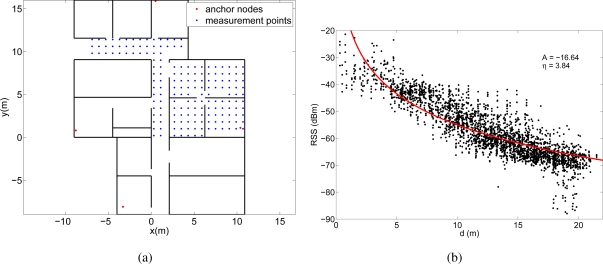
**(a)** Deployment areas in the WiFi experiment. **(b)** Experimental RSS measurements (689*4 values) for different distances between the PDA and the WiFi access points. The lognormal channel model curve fitting is also represented.

In the first experiment, we deployed four WiFi access points (Aruba AP-65) in the testing area and we measured the RSS with a PDA (HP iPAQ hw6915) in various points following a 80 cm × 80 cm grid. Figure 6(a) shows the deployment area with the position of the access points and the measurement points. In each of the measurement points we took 4 different RSS measurements from each access point, one for each orientation of the person who held the PDA (north, east, west and south). These four measurements were considered as different test positions to be localized separately. Altogether 689 RSS were taken from each access point. The transmission power of the WiFi access points was set to 20 dBm and their coverage was big enough to cover almost all the testing area. Therefore, at most of the measurement points signal was received from the four access points and, in the worst case, at least three access points were heard.

**Figure 7.**
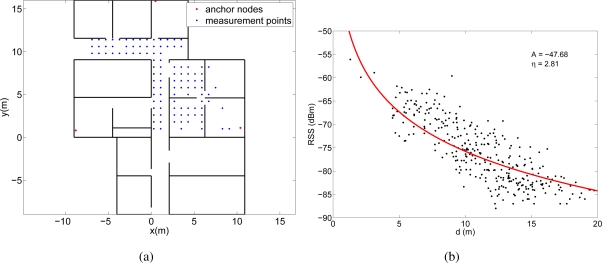
**(a)** Deployment areas in the Bluetooth experiment. **(b)** Experimental RSS measurements (107 values) for different distances between the PDA and the Bluetooth access points. The lognormal channel model curve fitting is also represented.

In the second experiment, we deployed three Bluetooth access points (minicomputers with a Belkin F8T013xx1 Bluetooth USB adapter) in the same office area and we measured the RSS from a PDA (HP iPAQ hw6915) that was situated in various points following the same grid as in the WiFi experiment. Figure 7(a) shows the deployment area with the position of the access points and the measurement points. All the Bluetooth devices were Class II devices, so their maximum transmission power is 4 dBm and their coverage is around 10 m. The total number of measurement points from which we were able to measure the RSS at the three access points simultaneously was 107 (see Figure 7(a)).

**Figure 8.**
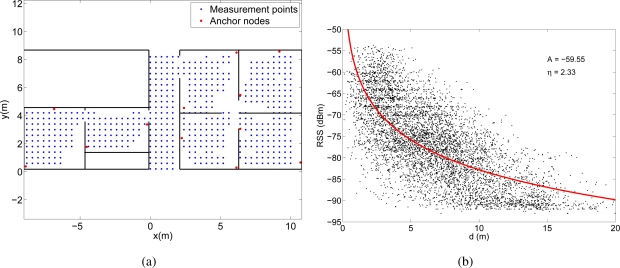
**(a)** Deployment areas in the WSN experiment. **(b)** Experimental RSS measurements (2795 values) for different distances between the mobile node and the anchor nodes. The lognormal channel model curve fitting is also represented.

Finally, in the third experiment, we deployed a MicaZ sensor network composed of twelve anchor nodes situated at fixed positions, five mobile nodes mounted on a platform that moved across the deployment area and a base node connected to a PC. The mobile nodes measured at every position the RSS from each anchor node and sent these measurements to the base, in order that the PC performed the RSS-distance conversion and the positioning algorithm. As the five nodes were situated at slightly different positions on the platform (they were separated between 15 and 30 cm), their measurements were considered as different test positions to be localized separately. Figure 8(a) shows the position of the anchor nodes and the 559 measurement points in the area of deployment. In each of the these points we took RSS measurements with the five mobile nodes, so altogether 2795 RSS measurements were taken from all the available anchor nodes. The transmission power of the MicaZ devices was set to 0 dBm so their coverage was around 6 m, depending on the walls and furniture. Therefore, the number of anchor points from which we took the RSS was different depending on the position of the mobile node, but in all the cases, we took at least RSS measurements from 3 anchor nodes. The high beacon density of this deployment comes from the fact that we must guarantee coverage from at least three reference nodes in all the area in order to be able to perform the localization.

The three deployments work in the 2.4 GHz ISM band and with real propagation conditions, that is, people moving in the office, walls and furniture that act as obstacle for the radio signal and produce non-line-of-sight propagation, *etc*. Although the deployment area is not very wide, the conclusions obtained from the results of these experiments can be extrapolated to bigger setups, as the performance of the positioning algorithms actually depends on the number of beacons that the mobile node can see (and their position), rather than on the size of the deployment area. This number of beacons depends itself on the density of beacon nodes and on the coverage radio, and is usually small in real applications (but at least 3, to guarantee localization). We have chosen the experiments to have different beacon densities and different coverage areas, but always a realistic number of nodes heard by the mobile node (*n*): a very low value in the Bluetooth and WiFi experiments (3 and 4, respectively) and a higher value in the wireless sensor network experiment (between 5 and 12 depending on the zone, 10.8 in average).

### 7.2. Estimated Channel Models

These RSS measurements were then converted into distances through the lognormal channel model in Equation (1). Obviously, the channel behavior is different for each experiment, so we estimated a different channel model for each case. To obtain the parameters *A_estim_* and *η_estim_* of the models we represented the RSS measurements obtained in each experiment as a function of the distance between the measurement point and the anchor node. Then we fitted the model in Equation (1) to these data using the Levenberg-Marquardt algorithm. Figures 6(b), 7(b) and 8(b) show the fittings and the estimated values of the parameters *A* and *η* for the three experiments, respectively.

The received signal strengths in the WiFi experiment are quite high, since the transmission power of the WiFi access points was set to 20 dBm. However, in the other two experiments (Bluetooth and MicaZ) the environment was not so trivial, as the power levels were lower and often close to the sensitivity of the receivers.

The differences between the estimated values of *A* and *η* for the three experiments is due to several reasons. First, the value of *A* depends on the transmission power, the antenna gains and the propagation loss at a distance of 1 meter. Therefore, the values are different for the three experiments because the transmission power and the antenna gains were different. For example, the transmission power for the WiFi devices was 20 dBm, whereas for the MicaZ devices was 0 dBm. Second, the lognormal model was fitted to the experimental data to calculate at the same time *A* and *η*. As it can be seen in Figures 6(b), 7(b) and 8(b) the RSS measurements for the WiFi deployment are quite above the sensitivity of the receiver, whereas for the MicaZ and Bluetooth deployments many measurements are lost due to the sensitivity threshold. Therefore, in the MicaZ and Bluetooth cases, the lognormal curve fitted to this incomplete set of measurements is flatter than expected so the estimated values of *A* and *η* are lower.

### 7.3. Localization Accuracy and Processing Time

**Figure 9.**
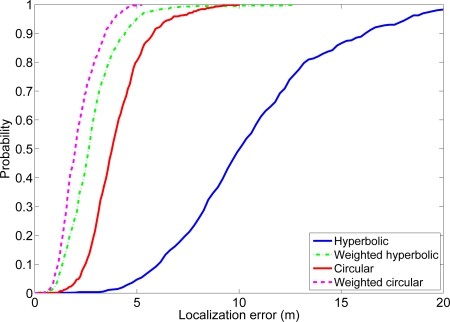
Cumulative distribution function of the localization error in the wireless sensor network experiment using the four positioning algorithms.

Finally, with the estimated distances, the proposed positioning algorithms were used to estimate the location of the PDA (in the WiFi and Bluetooth experiments) and the mobile nodes (in the sensor network experiment). For the weighted hyperbolic algorithm the final position is obtained using Equations (10), (8), (11) and (17). For the weighted circular algorithm, the final position is calculated by minimizing Equation (20) using a straight gradient method (Equation (3)). Again, the standard hyperbolic and circular positioning algorithms were also computed as a reference for comparison. Table 1 shows the average error and the average processing time of the four different techniques for the three experiments. Figure 9 shows, as an example, the cumulative distribution function (CDF) of the positioning error for the wireless sensor network experiment. The CDFs for the Bluetooth and the WiFi experiments show a very similar behavior, with the different positioning algorithms in the same order as in the wireless sensor network CDF.

As it can be noticed, the three experiments validate what we expected from theory and observed in the simulations: the weighted hyperbolic positioning technique has a better performance than the hyperbolic positioning technique and the weighted circular positioning technique has a better performance than the circular positioning technique. Again, we can also observe, as we did in the simulations, that the weighted circular positioning technique has the best performance and that the weighted hyperbolic positioning technique has a better performance than the circular positioning technique. This is a remarkable fact because this least squares technique also has a lower computational load. Therefore, this algorithm shows a good compromise between localization accuracy and computational cost.

### 7.4. Robustness to Channel Model Estimation Errors

In the experiments we described, we have used the model that was fitted to the experimental data for the RSS-distance conversion. But in a real localization situation, this is not always possible; the channel is usually characterized from a collection of previous measurements and would be different from the one we have used. Therefore, from the practical point of view, it has a great interest to evaluate the sensitivity of the proposed algorithms to variations in the estimated channel parameters, as we did with the simulations.

**Figure 10.**
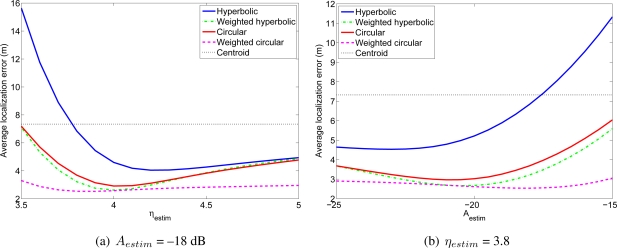
Average localization error in the WiFi experiment for different values of *η_estim_* and *A_estim_*.

**Figure 11.**
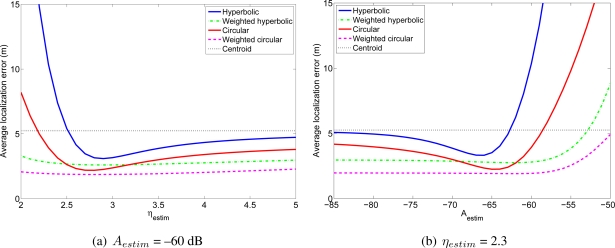
Average localization error in the wireless sensor network experiment for different values of *η_estim_* and *A_estim_*.

Figure 10(a,b) shows the average localization error in the WiFi experiment for different values of the lognormal channel model parameters. And Figure 11(a,b) shows the average localization error in the wireless sensor network experiment. The results for the Bluetooth experiment are not included because they are very similar to the other two. As a reference, we have included in these figures the localization errors of the centroid positioning technique, which estimates the position of the mobile node as the centroid of the positions of the reference nodes from which the mobile node receives signals.

In the WiFi and in the wireless sensor network experiments it can be seen that the two proposed algorithms have better accuracy than the corresponding non-weighted algorithms for any value of the parameters of the channel model. That is, the weighted hyperbolic positioning algorithm always gives better localization results than the standard hyperbolic algorithm and the weighted circular algorithm always gives better localization results than the standard circular algorithm. Furthermore, when the channel model differs from the optimum one (the one for which the localization errors are the lowest, which corresponds in the WiFi case for example to *η_estim_* between 3.8 and 4.3 and *A_estim_* between −22 dB and −17 dB, depending on the algorithm), the improvement when using the proposed positioning techniques may be quite significant. Usually, the parameters of the channel model are estimated from a group of measurements or even theoretically, so in general, the proposed algorithms will lead to better localization results. As an example, the estimated channel model for the WiFi experiment had parameters *η_estim_* = 3.839 and *A_estim_* = −16.64 dB. In this case, the localization error for the hyperbolic positioning algorithm is 7.73 m, whereas for our weighted methods the average errors are 3.69 m and 2.56 m.

## 8. Conclusions

In this paper we have proposed the use of two weighted positioning algorithms to calculate the position of a mobile node in an ad hoc network from a set of distance estimations to the anchor nodes. We have extensively proved through numerical simulations and real experiments that these algorithms provide better localization results than the traditional hyperbolic and circular positioning algorithms, as they consider the accuracy of each distance estimation. Furthermore, we have seen that this improvement is especially important when the estimation of the channel model is not very accurate, which means that the proposed algorithms are more robust to inaccuracies in the channel estimation. This fact makes the algorithms very attractive for real localization applications in which the channel model must be estimated from a limited set of measurements, or cannot be calibrated at all.

In terms of accuracy, the circular algorithms perform better than the hyperbolic algorithms, as in the second case the cost function to be minimized is a non-linear function of the error. However, due to their non-iterative nature, the hyperbolic algorithms have the advantage of a lower computational cost (in the weighted hyperbolic algorithm this is true for small values of *n*, which is the usual case in real deployments). Note, however, that in the circular algorithms we have used a gradient search method for minimizing the error functions. Other minimization techniques with lower convergence time could be applied, yielding better results in terms of computational cost. In any case, in order to choose between one or other technique, the accuracy-cost trade off must be considered together with the application characteristics.

For example, in terms of computational cost, the cost of the weighted hyperbolic technique is only slightly higher than the cost of the hyperbolic algorithm, and lower than the cost of the circular algorithm for the typical characteristics of real deployments. Therefore, the use of this algorithm will increase the accuracy of the localization system, at the expense of a little increase in the computational cost. Again, in a real deployment, where the channel estimation may not be very accurate, the use of this positioning algorithm may definitively improve the localization system performance. The weighted circular technique would be useful in applications that need a great accuracy in the localization and do not have energy restrictions or temporal constraints.

In further work we are planning to empirically evaluate the computational cost of these algorithms in terms of energy consumption. In particular, we have recently implemented embedded versions of the four algorithms (in MicaZ motes, under TinyOS 2.1) to evaluate with real-field experiments the energy consumption during the localization when the algorithms are executed in these resource-constrained devices. Preliminary results show, as otherwise expected, that whereas circular algorithms are more suitable for applications in which the localization is done occasionally, the weighted hyperbolic technique is appropriate and practical for real-time location tracking, as its computation time is very short and almost identical to that of the classical hyperbolic technique.

## Figures and Tables

**Table 1.** Average localization error and average processing time for the different positioning algorithms in the three experiments.

WiFi (N = 4)				

	Hyperbolic	Weighted hyperbolic	Circular	Weighted circular
	
Average localization error	7.73 m	3.69 m	4.06 m	3.03 m
Average processing time	0.04 ms	0.11 ms	2.24 ms	4.04 ms

Bluetooth (N = 3)				

	Hyperbolic	Weighted hyperbolic	Circular	Weighted circular
	
Average localization error	4.58 m	4.58 m	3.93 m	2.70 m
Average processing time	0.04 ms	0.10 ms	2.44 ms	3.02 ms

WSN (N = 12)				

	Hyperbolic	Weighted hyperbolic	Circular	Weighted circular
	
Average localization error	10.59 m	2.80 m	4.01 m	2.14 m
Average processing time	0.03 ms	0.13 ms	1.48 ms	3.53 ms
